# The Relationship Between “Protect People's Livelihood” and “Promote the Economy:” Provincial Evidence From China

**DOI:** 10.3389/fpubh.2021.722062

**Published:** 2021-08-02

**Authors:** Baicheng Zhou, Shu Wang, Zhi Qiao

**Affiliations:** ^1^China Center for Public Sector Economy Research, Jilin University, Changchun, China; ^2^School of Economics, Jilin University, Changchun, China

**Keywords:** healthcare expenditure, economic growth, time-varying, transmission channel, China

## Abstract

This study focused on medical care in a single country (China) and in regions with different economic backgrounds and different economic development levels to determine the effect of healthcare expenditure on short- and long-term economic growth. The study supported some interesting conclusions: (1) For most areas of China, increasing healthcare expenditure has a negative impact on economic growth in the short term but promotes growth in the long run; (2) Under different levels of economic development within China, there is significant heterogeneity in the interaction between healthcare expenditure and economic growth; (3) The negative effects of healthcare expenditure on short-term economic growth are greater during periods of economic turbulence than during times of stability; and (4) Healthcare expenditure has a negative effect on underdeveloped areas through the accumulation of material capital, while in economically developed areas, this channel has the opposite effect. To improve the quality of medical and health protection and the quality of life and welfare, China needs to consider the development characteristics of different economic zones and establish a multilevel, systematic and diversified medical and health protection system.

## Introduction

In early 2020, COVID-19 unexpectedly swept the world, with more than 80 million people diagnosed and nearly two million dead in less than a year. Governments worldwide launched a series of targeted measures to protect people's health and save their economies. However, it is not possible to both “protect people's livelihood” and “promote the economy.” If strict isolation is implemented to prevent the further spread of the virus, it will result in a costly sharp drop in income, a soaring unemployment rate and an economic downturn. Even countries that try to maintain economic activities with the expectation that mass immunization will take effect may find that this strategy does little to maintain the normal operation of the economy. From a global perspective, with the advent of autumn and winter, the number of infected people has not decreased. Instead, the new coronavirus has mutated. The pain brought by COVID-19 may continue to affect human society well into the future. China was the first country to confront this new epidemic, but it also took the lead in restoring economic order under the epidemic, which was closely related to the anti-epidemic disaster relief provided by the government for the Chinese people regardless of the cost. China has made great efforts in the fight against COVID-19. On June 7, 2020, the *White Paper on China's Action against the Epidemic of Coronavirus Pneumoni*a published by the Press Office of the State Council of China showed that the average medical cost for confirmed patients nationally was ~23,000 yuan, and the treatment cost of individual critically ill patients reached up to millions of yuan. All of these costs were borne by the state, and the total expenditure was well-beyond 1 billion yuan. The interaction between healthcare expenditure and economic growth is one of the key areas of public health research in China. The sudden outbreak of COVID-19 and the resulting trade-off between “protecting people's livelihood” and “promoting the economy” increase the value of discussing the impact of healthcare expenditure on the market economy. Not only China, but the whole world is facing such a test, especially at the time when the COVID-19 epidemic is raging. Is it possible to balance “protecting people's livelihood” and “promoting growth” as much as possible, directly affecting the development of a country or region's medical and health security system, people's livelihood and welfare, and long-term economic development. For the whole world, the establishment of a multi-dimensional, three-dimensional, and targeted medical and health security system is of great practical significance.

A medical and health security system can provide people with better medical care, effectively improve the health status of residents, improve life expectancy, and increase social welfare and overall safety (1, 2). However, its impact on economic growth is still subject to debate. There may be differences in the economic growth effect of healthcare expenditure given different economic contexts, and the path through which healthcare expenditure influences economic growth is not clear. Countries with different medical insurance systems have various consumption behaviors and investment concepts, and so they experience diverse effects of healthcare expenditure on economic growth, which is a topic of broad interest (3–7). However, the heterogeneity of the effect of healthcare expenditure on economic growth in distinct regions of the same country has not been discussed in depth. In a country like China, in particular, with its vast land area and great differences in development between the north and the south, different economic zones will have distinct the economic structures (8–13). Therefore, the impact of healthcare expenditure on economic growth is also likely to differ. Further comparative analysis is needed to reveal the complex mechanism through which economic growth affects healthcare expenditure in each economic zone within the same country.

From the above analysis, it can be seen that previous studies have discussed the effect of health expenditure on economic growth from various perspectives and reached many meaningful conclusions. However, there are still several research gaps to be filled. The previous literature generally studied the economic growth effect of healthcare expenditure based on the constant parameter method, but, in fact, there should be differences in the impact of healthcare expenditure on economic growth against different economic contexts, and the constant parameter model cannot describe this subtle variety. Previous research that adopted an econometric model to identify the economic growth effect of healthcare has been able to partially identify the different impacts of health expenditure on economic growth among countries with diverse economic systems but has not paid sufficient attention to the same differences in impacts across different development regions within a country. Most of these studies treat the impact of domestic healthcare expenditure on regional economic growth as homogeneous, and they do not explore the dynamic trajectory of the impact of healthcare expenditure on economic growth in different economic zones. In addition, there is no research that incorporates the numerous economic variables involved in the process through which healthcare expenditure acts on economic growth into the systematic analysis framework. The lack of important influencing variables may lead to a large deviation between the analysis results and the real economy, which is another reason why many studies fail to reach a unified conclusion on whether healthcare expenditure drives or inhibits economic growth.

In view of this gap, this paper attempts to construct a non-linear model of the dynamic time-varying relationship between healthcare expenditure and economic growth in China under a systematic analysis framework with a large number of possible economic variables and then empirically identifies the impact of medical and health spending on economic growth in China and in its distinct domestic economic regions, analyzing the possible transmission path for the impact of healthcare expenditure on economic growth. Compared with the existing literature, the marginal contribution of this paper is as follows. First, an econometric model with time-varying parameters is constructed to identify the effect of healthcare expenditure on economic growth to effectively capture whether healthcare expenditure stimulates or inhibits economic growth given different economic contexts in different periods. We also identified and distinguished the economic growth effect of healthcare expenditure in China nationally and regionally, which provides empirical evidence for the differential and comprehensive improvement in China's medical security system. Second, this paper provides not only an effectively analysis of the dynamic adjustment mechanism behind healthcare expenditures' impact on economic growth but also discusses the path through which healthcare expenditure impacts economic growth under a systematic analysis framework including a broad set of possible economic variables. This work offers a new understanding of the transmission characteristics for the effect of medical care and health on economic growth and can be regarded as a critical supplement to current research on the improvement in the medical security system.

The rest of this document is arranged as follows: the second section offers a literature review and develops the hypotheses. The third section constructs the time-varying parameter measurement model and introduces the selection of variables and data description. The fourth section analyzes the economic growth effect of healthcare expenditure, discusses the influence and transmission path between China's health expenditure and economic growth in different economic areas, and conducts a robustness test. The last section summarizes the full work and provides policy recommendations.

## Literature Review and Hypothesis Development

As a vital guarantee of national livelihood security, the medical security system plays a stabilizing role in economic development. During rapid economic development, the government can increase medical security expenditure on a large scale and improve medical security treatment to buffer the speed of economic development; when the economy is at a low, medical security expenditure can replace residents' preventive savings and stimulate domestic demand to encourage residents' consumption. In the past, most studies on the relationship between expenditure and economic growth have focused on the macro perspective (14–16). Some scholars deem that the rise of medical security expenditure will directly affect the consumption and investment tendencies of employees, effectively guarantee the quality of human capital (17), prolong life expectancy (18), and reduce infant mortality (19) to improve the savings rate, play a positive role in the growth of per capita income, and positively facilitate economic advance. However, the research of other scholars supports the completely opposite conclusion. Zhu and Hu (7) believe that healthcare expenditure will produce a negative effect on economic growth, which can be suppressed by enhancing the coverage rate of the medical security system. Likewise, Yang et al. (20) established an endogenous growth model and found that health expenditure would inhibit economic growth. According to the literature on China's health expenditures as well as economic growth, for low-income families, coverage of the medical security system is an effective guarantee of consumption. Although medical security consumption augments household expenses, it has a significantly promoting function on non-medical consumption and, in this way, influences economic growth. Tao and Wang (21) divided Chinese government health expenditure into four categories—medical and health service expenditure, medical security expenditure, administrative affairs expenditure and population and family planning affairs expenditure—and considered that only government medical security expenditure is able to promote positive economic growth. Accordingly, the first set of opposing hypotheses explored in this paper is established:

Hypothesis 1a. China's healthcare expenditure has a positive effect on economic growth.
Hypothesis 1b. China's healthcare expenditure has an inhibitory effect on economic growth.

Although the direction of medical and health spending on economic growth is indeterminate, a considerable number of studies have discussed this issue. In contrast, the specific mechanism through which healthcare expenditure effects economic growth has been ignored. Representative research results show that the economic growth effect from healthcare expenditure is mainly realized through the accumulation of material and human capital. Specifically, healthcare expenditure will occupy household consumption expenditures and directly affect the consumption allocation of residents. Based on precautionary savings theory, this behavior will raise household consumption and reduce savings as well as the speed of material capital accumulation (22). As a result, economic growth is slowed; however, the medical insurance system can avoid the poverty caused by illness to a certain extent and stabilize income expectations by reducing the economic risk of residents. Numerous scholars have also proven that the medical security system is a “necessity” rather than a “luxury” for most countries from mathematical and empirical perspectives (23), while the medical security system can enhance personal savings during the working span of the labor force (24, 25). Thus, it produces a positive impact on economic growth. In other words, the direction of the impact of material capital accumulation on medical expenditure on economic growth is uncertain. The research conclusions regarding the effect of human capital accumulation on healthcare expenditure and thereby on economic growth are relatively uniform. Zhu et al. (26) and Jia et al. (27) and other scholars deem that the increase in healthcare expenditures can not only reduce the newborn mortality rate and prolong the average life span but also increase investment in health and educational capital by increasing leisure time through the substitution effect, which can play a positive role in promoting the economic growth effect of healthcare expenditure. The relationship between material capital accumulation, human capital accumulation and health expenditure on economic growth can be summarized as follows.

China is a developing economy: the medical security system is not perfect, the population is large, and a considerable part of the population has little disposable income. Therefore, in the short term, healthcare expenditure creates real pressure on middle- and lower-level families, further reducing their disposable income and thereby affecting economic growth. A long-term medical insurance system can effectively alleviate the family poverty caused by illness, providing a significant umbrella for ordinary families in the face of natural and man-made disasters and preserving the time and energy of residents for investment in human capital reserves, so it may have a positive impact on economic growth. Based on this reasoning, the second assumption of this paper is proposed:

Hypothesis 2. China's healthcare expenditure plays a role in economic growth through the accumulation of material and human capital. The material accumulation channel has a negative impact on the economic growth effect of healthcare expenditure in the short term and a positive effect in the long term. The human capital accumulation channel has a positive impact on the economic growth effect of healthcare expenditure in both the short and long term.

Few researchers have also attempted to seek the mechanism through which medical security expenditure affects economic growth considering aspects such as residents' consumption, aging, degree of economic openness, employment rate, economic development level, etc. (28–32). Taking into account the imbalance in regional economic development in China, healthcare expenditures in different regions are also significantly different. Merely considering the economic growth effect of China's healthcare expenditure from a national perspective will not provide a comprehensive understanding; therefore, the last assumption of this article is proposed:

Hypothesis 3. The economic growth effect of China's healthcare expenditure is significantly different in different regions.

## Methods and Data Sources

To test hypotheses 1, 2, and 3, this paper designs the following research process.

### Data Sources

This paper focuses on the effect of China's healthcare expenditure on economic growth and holds that healthcare expenditure will have different effects on long- and short-term economic growth. The impact of healthcare expenditure on economic growth is regulated by the medical security system and is mainly realized through the accumulation of material and human capital. In this study, China's healthcare expenditure is represented by the proportion of healthcare expenditure in GDP (%), short-term economic growth is represented by the quarterly per capita GDP growth rate (%), and long-term economic growth is represented by the 5-year per capita GDP growth rate (%). When discussing the transmission path for the effect of medical expenditure on economic growth, the accumulation of physical capital and human capital are, respectively represented by the total amount of fixed assets investment and the percentage of education expenditure in GDP (%). The specific data are described in Table 1.

**Table 1:** Statistical description of the main variables in the article.

**Var**.	**Var. Def**.	**Sam. Per**.	**Mean**	**Max**.	**Min**.	**Std. Dev**	**Median**
HCE_CN	Healthcare expenditure in GDP (%)	2003Q1–2019Q4	4.503	7.545	0.652	0.233	4.766
GDP_S	China's quarterly per capita GDP growth rate (%)	2003Q1–2019Q4	3.004	5.611	1.548	0.130	2.607
GDP_L	China's 5-year per capita GDP growth rate (%)	2003Q1–2019Q4	12.598	17.707	8.300	0.388	12.575
PCA	Physical capital accumulation expenditure in GDP (%)	2003Q1–2019Q4	57.806	1.271	0.112	52.578	0.714
HCA	Human capital accumulation expenditure in GDP (%)	2003Q1–2019Q4	18.674	22.670	15.428	0.223	18.433

*GDP, represents China's gross domestic product; Sam. Per., represents sample period; Var., variable; Max., maximum; Min., minimum; Std. Dev., standard deviation*.

In addition, considering that the effect of healthcare expenditure on economic growth will be influenced by many other economic factors, if the many economic variables potentially involved are not included in the systematic analysis framework, the empirical research results may deviate from the actual economic situation. Considering the latent factors that may affect the relationship between health expenditure and economic growth and the availability of data, this paper identifies and classifies 78 influencing factors, mainly related to the level of economic development, macroeconomic status, economic openness degree, population aging and so on. All data are quarterly data from the Wind database. The obvious seasonal factors are processed by X-12, the non-stationary data are processed by the ADF test, and some missing data need to be supplemented by interpolation.

### Estimation Method: SV-TVP-FAVAR Model

All the factors that may impact the economic growth effect of healthcare expenditure are included in the *Database*_*t*_. Concurrently, to eliminate the curse of dimensionality caused by including too many variables in the traditional model, we use the method of Bernanke and Boivin (33) to extract the data in the background dataset into several common factors that cannot be observed. The method is as follows:

(1) $$Database_{t}=\Lambda^{\bar{ob}}\bar{ob_{t}}+\Lambda^{\bar{un}}\bar{Un_{t}}+\varepsilon_{t}$$

where $\Lambda^{\bar{ob}}$ and $\Lambda^{\bar{un}}$ are factor loading matrices. $\bar{ob_{t}}$ and $\bar{un_{t}}$ are the observable part and the unobservable part, respectively. $\varepsilon_{t}^{\sim }N(0,\Omega_{t})$. Abstracting a large amount of information into several unobservable common factors can effectively avoid the problem of missing key data variables. However, if the economic growth effect of China's healthcare expenditure is calculated by the classical VAR model (34), it will be limited by the constant parameters of the model, with the exception of the degrees of freedom. A constant parameter model means that no part of the economic system can vary with time, and this would make it difficult to capture the effect of medical expenditure policy on economic growth in distinct economic periods. Therefore, in the above FAVAR framework, the parameters in the model are further extended to the time-varying mode:

(2) $$\{\begin{array}{l} x_{it}={\tilde{\lambda }}_{i}^{f}f_{t}+{\tilde{\lambda }}_{i}^{l}l_{t}+{\tilde{\lambda }}_{i}^{w}w_{t}+\zeta_{it}\\ y_{t}=b_{1t}y_{t-1}+\ldots +b_{pt}y_{t-p}+\xi_{t} \end{array}$$

${\tilde{\lambda }}_{i}^{f}$${\tilde{\lambda }}_{i}^{l}$ and ${\tilde{\lambda }}_{i}^{w}$ are the dynamic factor loading matrices in turn, and *f*_*t*_ is the extracted common factor, whose dimension is defined as (3 × 1)^1^; [*l*_*t*_, *w*_*t*_] is composed of long- and short-term economic growth rate and health expenditure as observation variables and proxy variables, respectively.

i=1,…,p, *t* = 1, …, *T*,$\xi_{t}^{\sim }N(0,\Omega_{t})$,$\zeta_{it}^{\sim }N(0,\exp(h_{it}))$.

Furthermore, let the residuals in **Equation (2)** be random walks; then, by solving the equation, we can obtain:

(3) $$x_{t}=\lambda^{f}f_{t}+\lambda^{l}l_{t}+\lambda^{w}w_{t}+\Gamma \left(L\right)x_{t}+\varepsilon_{t}$$

Γ(*L*) = *diag*(ρ^1^(*L*), …, ρ^*n*^(*L*)), $\rho^{i}\left(L\right)=\rho_{i1}L+...+\rho_{iq}L^{q}$;$\lambda^{j}=\left(I_{n}-\Gamma \left(L\right)\right){\tilde{\lambda }}^{j}$,*j* = *f, l, w*;$\varepsilon_{t}^{\sim }N(0,H_{t})$,*H* = *diag*(exp(*h*_1*t*_), …, exp(*h*_*nt*_)). The form of residuals with the random walk is $h_{it}=h_{it-1}+\eta_{t}^{h}$,$\eta_{t}^{h\sim }N(0,\sigma_{h})$. Thus, the SV-TVP-FAVAR model is constructed and used to analyze the economic growth effect of China's healthcare expenditure.

### Model Construction

Using the above framework to verify the first set of hypotheses and making full use of the advantages of MATLAB high-dimensional computing, we can draw the three-dimensional impulse response diagram for the effect of China's health expenditure on economic growth. Further exploring the transmission channel for the economic growth effect of health expenditure to test hypothesis 2, we can construct the following regression equation:

(4) $$\begin{array}{l} \;\left[\;\begin{array}{c} I_{short}\\ I_{long} \end{array}\right]=\left[\begin{array}{cc} \beta_{short,t}^{K} & \;0\\ 0 & \;\beta_{long,t}^{L} \end{array}\right]\;\left[\;\begin{array}{c} F_{K}\\ F_{L} \end{array}\right]\\ +\left[\begin{array}{cc} \beta_{short,t}^{L} & \;0\\ 0 & \;\beta_{long,t}^{K} \end{array}\right]\;\left[\;\begin{array}{c} F_{L}\\ F_{K} \end{array}\right]+\left[\;\begin{array}{c} e_{t}^{short}\\ e_{t}^{long} \end{array}\right] \end{array}$$

*I*_*short*_ and *I*_*long*_ are the short- and long-term economic growth effects of health expenditure, respectively; *F*_*K*_ and *F*_*L*_ are the effects of material capital accumulation and human capital accumulation, respectively; $\beta_{short,t}^{K}$, $\beta_{short,t}^{L}$, $\beta_{long,t}^{K}$ and $\beta_{long,t}^{L}$ are the respective time-varying coefficients of physical capital accumulation and human capital accumulation in the effect of health expenditure on economic growth. Further referring to Primiceri (35) and Nakajima et al. (36), we can divide ${\left[\begin{array}{cc} e_{t}^{short} & \;e_{t}^{long} \end{array}\right]}^{\prime }$ into lower triangular matrix A, diagonal matrix Σ and white noise sequence δ_*t*_:

(5) $$\begin{array}{l} \left[e_{t}^{k}\right]=\alpha^{-1}\sigma \delta_{t}={\left[\begin{array}{lll} 1 & \cdots & 0\\ \vdots & \ddots & \vdots \\ a_{k1} & \cdots & 1 \end{array}\right]}^{-1}\left[\begin{array}{lll} \sigma_{1} & \cdots & 0\\ \vdots & \ddots & \vdots \\ 0 & \cdots & \sigma_{k} \end{array}\right]\delta_{t} \end{array}$$

Following **Equation (3)**, let the model have the form of random fluctuation, and all the residuals conform to the innovative random walk. Then, this model can verify how the leading factors in the effect of health expenditure on economic growth change under diverse economic contexts. If hypothesis 2 is true, $\beta_{short,t}^{K}$, $\beta_{short,t}^{L}$, $\beta_{long,t}^{K}$, and $\beta_{long,t}^{L}$should be time-varying, and their strength will depend on the economic context. If the short-term impact of health expenditure on economic growth is mainly negative through the channel of material capital accumulation, and the long-term impact on economic growth is mainly positive through this channel, then there should be $\beta_{short,t}^{K}<0$,$\beta_{short,t}^{K}>0$. If, through the accumulation of human capital, the effect of health expenditure on economic growth is positive in both the long term and the short term, then $\beta_{short,t}^{L}$, $\beta_{short,t}^{L}>0$.

In reality, China possesses a vast territory, and its economic development gap between different regions is large. To comprehensively test hypothesis 3, considering China's economic areas and data availability, we select Beijing, the capital of China; Tianjin, a municipality directly under the central government in northern China; Jilin, a representative in the northeastern part of the old industrial base; Sichuan, a representative area in southwestern China; and Inner Mongolia, a representative in the Yellow River basin, to fit the impact of health expenditure in each region on the short- and long-term growth of China's economy and transmission channels. For a description of the economic data selected in this paper, please refer to Table 2. If hypothesis 3 is true, $I_{short/long}^{EZ}=\left[I_{NMG},I_{JL},I_{SC},I_{BJ},I_{TJ}\right]$ should be time-varying and distinct from each other. $\beta_{short/long,t}^{EZ,K}=\left[\beta_{NMG,t}^{K},\beta_{JL,t}^{K},\beta_{SC,t}^{K},\beta_{BK,t}^{K},\beta_{TJ,t}^{K}\right]$ and $\beta_{short/long,t}^{EZ,L}=\left[\beta_{NMG,t}^{L},\beta_{JL,t}^{L},\beta_{SC,t}^{L},\beta_{BK,t}^{L},\beta_{TJ,t}^{L}\right]$ will also show different characteristics due to the difference in economic range.

**Table 2:** Statistical description of China's healthcare expenditure in different regions.

**Var**.	**Var. Def**.	**Sam. Per**.	**Mean**	**Max**.	**Min**.	**Std. Dev**	**Median**
HCE_NMG	Inner Mongolia's proportion of healthcare expenditure in GDP (%)	2003Q1–2019Q4	0.996	1.817	0.174	0.056	1.019
HCE_JL	Jilin Province's proportion of healthcare expenditure in GDP (%)	2006Q1–2019Q4	1.383	2.720	0.147	0.081	1.330
HCE_SC	Sichuan Province's proportion of healthcare expenditure in GDP (%)	2006Q1–2019Q4	1.564	2.241	0.160	0.072	1.701
HCE_BJ	Beijing's proportion of healthcare expenditure in GDP (%)	2009Q1–2019Q4	1.409	1.610	0.366	0.033	1.444
HCE_TJ	Tianjin's proportion of healthcare expenditure in GDP (%)	2011Q1–2019Q4	0.689	1.271	0.112	0.048	0.714

*HCE, represents China's healthcare expenditure in different regions; Sam. Per., represents the sample period; Var., variable; Max., maximum; Min., minimum; Std. Dev., standard deviation*.

## Empirical Results

### Unit Root Test and Selection of Optimal Lag Order

Before conducting empirical research, it should be noted that all economic variables included in the systematic analysis framework should be handled as stable to avoid the problem of spurious regression. Table 3 provides the ADF test results for health expenditure in China, Inner Mongolia, Jilin, Sichuan, Beijing, and Tianjin, as well as the short- and long-term economic growth data of the corresponding regions, all of which are consistent with first-order stationary data. Similarly, the ADF test is carried out to measure the material capital accumulation and human capital accumulation of the possible transmission path of the economic growth effect of health expenditure in different economic regions, and the consequences are also put into Table 3. It can be seen that material capital accumulation and human capital accumulation in diverse economic regions of China are stable data after handling.

**Table 3:** The results of ADF unit root tests for healthcare expenditure (HCE) and GDP.

**Regions**	**HCE**		**GDP_S**		**GDP_L**	
	**Level**	**First difference**	**Level**	**First difference**	**Level**	**First difference**
CN	−1.609	−3.526^**^	−1.955	−8.366^***^	−2.804	−1.179^*^
NMG	−0.039	−4.433^***^	−1.401	−8.844^***^	−0.980	−8.326^***^
JL	−0.336	−6.520^***^	0.695	−8.144^***^	0.6334	−8.135^***^
SC	−2.361	−3.079^**^	−2.300	−8.614^***^	−2.018	−8.081^***^
BJ	−1.609	−4.496^***^	−1.867	−6.315^***^	−2.046	−8.128^***^
TJ	−1.492	−3.823^***^	0.462	−8.239^***^	1.462	−7.976^***^

*, **, or *** *denote that the null hypothesis is rejected at the 10, 5, or 1% significance levels; HCE, represents China's healthcare expenditure in different regions; GDP_S and GDP_L, represents China's short-term economic growth and long-term economic growth in different regions, respectively*.

### Empirical Results of the SV-TVP-FAVAR Model

**Figure 1:**
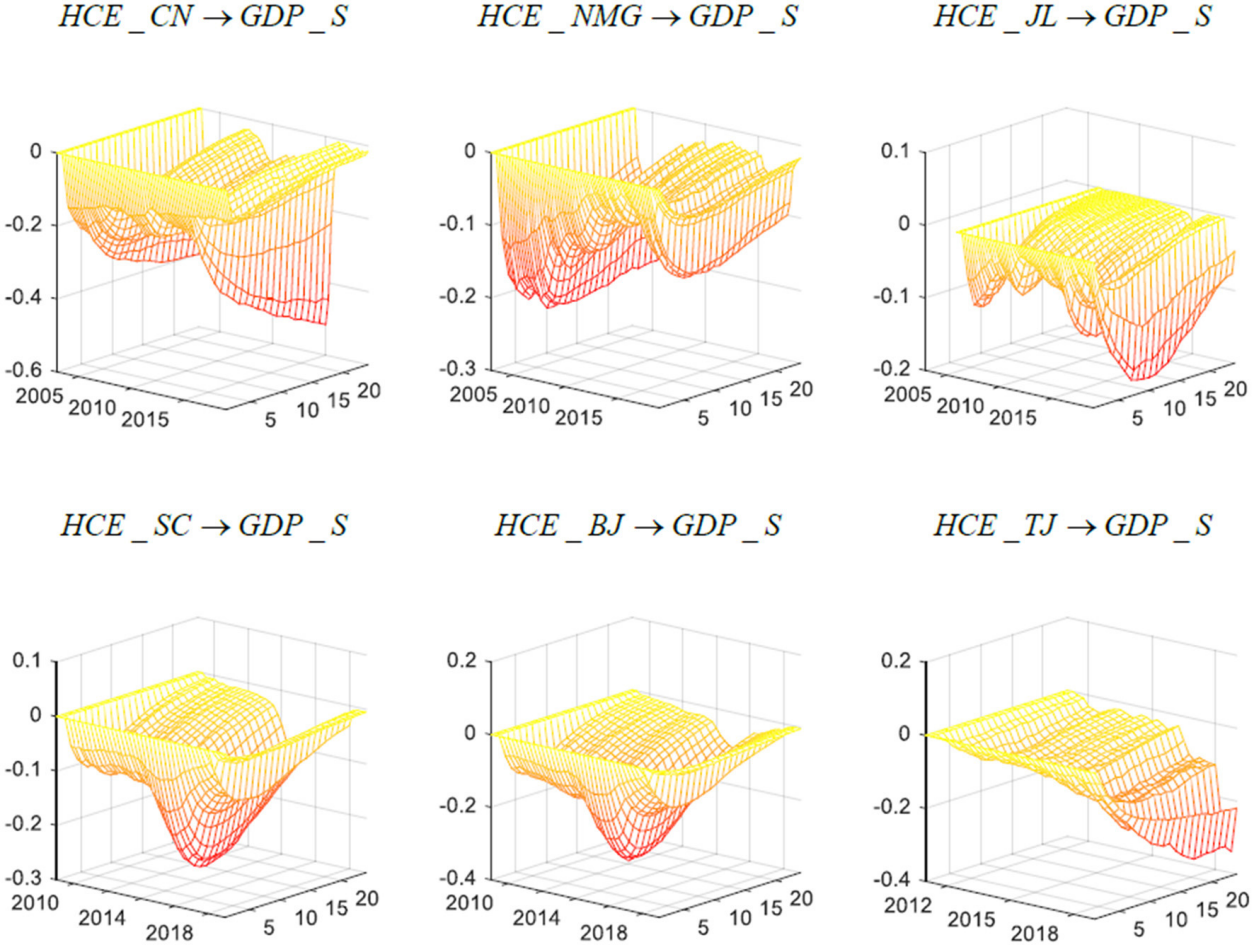
Three-dimensional impulse response of China's short-term economic growth in different regions. Each subfigure with the title of “*X* → *Y*” demonstrates the response of variable Y to an orthogonalized positive shock to variable X. In other words, X is an impulse variable, and Y is a response variable. One period in the figure denotes one season.

This section is based on the model in the previous section. First, the historical characteristics of the evolving effect of China's healthcare expenditure on economic growth are analyzed by the time-varying parameter method, and then the possible transmission path for the healthcare expenditure effect on short-term and long-term economic growth is discussed based on the pulse response method. Overall, the comprehensive combination of the dynamic change in the effect of China's healthcare expenditure on economic growth and its mechanism in recent years allows us to obtain some revealing findings as follows:

Figure 1 demonstrates three-dimensional impulse response diagrams of the impact of healthcare expenditure on short-term economic growth in China, Inner Mongolia, Jilin, Sichuan, Beijing and Tianjin. In this figure, the x-axis is the time dimension and reveals the time point of the impact of health expenditure on the economy in the short term; the y-axis is the response dimension, showing the duration of the impact of health expenditure on economic growth; and the z-axis indicates the strength of the effect of health expenditure on economic growth. From the results of the empirical analysis, health expenditure has a significant negative effect on short-term economic growth in China. Specifically, in the short term, an increase in the proportion of health expenditure will lead to a decrease in the short-term economic growth rate. From the time dimension analysis, it can be found that during the period of global economic crisis and the new normal of the Chinese economy, the impact of health expenditure on economic growth in China, except for Tianjin, has increased significantly, which shows that the public health insurance system further inhibits the speed of economic growth when the economic system is seriously impacted. This is consistent with the economic facts, since in the period of global economic crisis included in the sampling interval, a multitude of enterprises closed down, the economy fell into stagnation, and the unemployment rate rose abruptly. These negative factors all led to weak economic growth. China's entry into the “new normal” is similar. At this stage, facing a more complex and changeable domestic and international environment, China had a slower economic growth rate, and its economic development mode changed from “quantity” to “quality.” Therefore, in this economic context, enhanced health expenditure will undoubtedly increase the financial burden of the government and negatively impact short-term economic growth. Similarly, the impulse response results support the above inference. When China's economic system is affected by a negative external impact, the duration of health expenditure's impact on economic growth is extended, and the adverse effect of the increase in health expenditure on the speed of economic growth lasts longer during periods of economic fragility.

**Figure 2:**
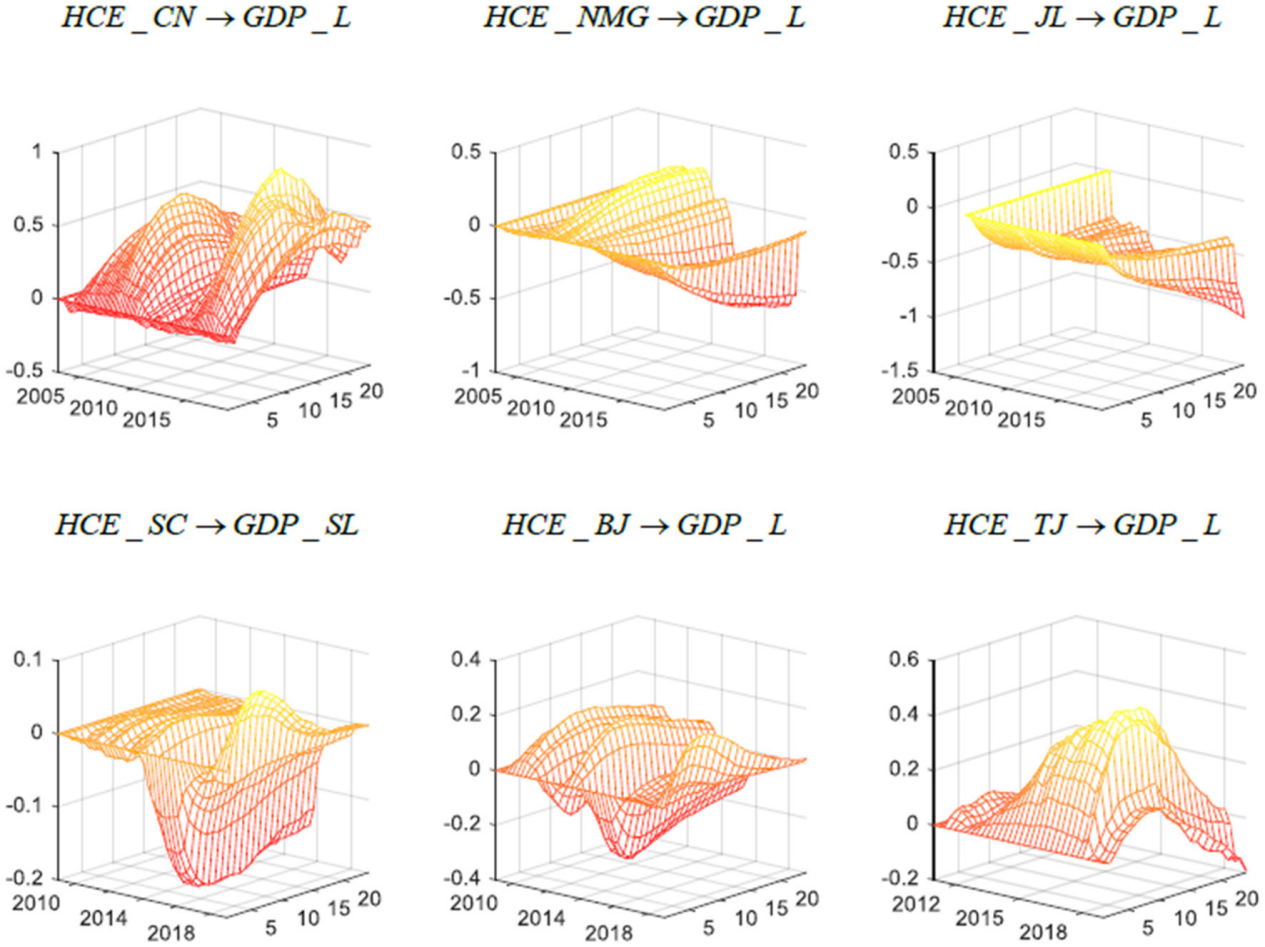
Three-dimensional impulse response of China's long-term economic growth in different regions. Each subfigure with the title of “*X* → *Y*” demonstrates the response of variable Y to an orthogonalized positive shock to variable X. In other words, X is an impulse variable, and Y is a response variable. One period in the figure denotes one season.

Figure 2 shows the impact of health expenditure on long-term economic growth. The x-axis and y-axis are the time dimension and response dimension, respectively, and represent the time point and duration of the impact of healthcare expenditure on long-term economic growth in China, while the z-axis is the three-dimensional impulse response amplitude of the economic growth effect of healthcare expenditure. In general, compared with the negative impact of health expenditure on short-term economic growth, the heterogeneity of health expenditure on long-term economic growth is more obvious, verifying hypothesis 3. The overall data of China show that healthcare expenditure will promote long-term economic growth. The reason may be that the establishment of the medical and health security system has greatly improved the situation of the overall population, thus increasing the effective labor supply and promoting economic growth through the accumulation of human capital in the long run. The reason may also be that from a nationwide perspective, the medical insurance system can effectively reduce the probability of poverty caused by illness and reduce the economic risk of residents, increasing personal savings in the long run and having a positive impact on economic growth by way of material capital. Through the in-depth analysis of the impact of health expenditure on long-term economic growth in diverse economic regions of China, it can be seen that improvement in health expenditure still resists long-term economic growth most of the time for Inner Mongolia, Jilin, and other regions, and this adverse impact on long-term economic growth shows an increasing trend in the time dimension. Because both inner Mongolia and Jilin are old industrial bases with similar economic structures and are among China's relatively underdeveloped areas, their main driving force for economic growth comes from the labor-intensive secondary industry. In recent years, the economic gap between northern and southern China has gradually widened, which has also led to the further slowdown of economic growth in Inner Mongolia and Jilin. The pressure of healthcare expenditures on local finances is more apparent in these two regions, which reduces the benefits of the improved medical and health security system. Sichuan is located in the hinterland of Southwest China and is rich in resources. In recent years, a group of urban areas led by Chengdu have developed rapidly, undertaking part of the spillover capacity in the Yangtze River Delta. The increase in healthcare expenditure in Sichuan can slightly stimulate economic growth during a period of stable economic growth but will have a certain negative impact on long-term economic growth as China's economy enters the new normal. After 2018, with the improving economic development of Sichuan Province, local finance can withstand the pressure of increasing healthcare expenditures. Therefore, the long-term positive effect of the medical and health security system on the economy is obvious, showing that the impact of healthcare expenditure on long-term economic growth has increased. Beijing is the capital of China, and it thus undertakes more administrative functions. The impact of healthcare expenditure will accelerate Beijing's long-term economic growth most of the time but cause a negative response to Beijing's long-term economic growth during a period of shifting national economic growth. Tianjin, one of the municipalities directly under the central government in China, is one of the core areas of the Beijing Tianjin Hebei Economic Zone, which undertakes part of the productivity transfer in Beijing. For Tianjin, healthcare expenditure will significantly promote local economic growth, and its long-term effect on economic growth has an increasing trend over time, which indicates that the advantages of healthcare expenditure on the long-term economic growth of Tianjin are greater than the disadvantages. The results are similar to the economic growth effect of national healthcare expenditure.

In conclusion, the results of the three-dimensional impulse response function illustrate the impact of health expenditure on short- and long-term economic growth and offer an initial exploration of the possible causes of heterogeneity in the effects of medical and health expenditures on economic growth in different economic contexts and different economic regions. The empirical results show that hypothesis 1 is incomplete. In the short run, increasing health expenditures will have a negative impact on economic growth, while in the long run, the benefits of increasing health expenditure gradually emerge, as reflected in the acceleration of long-term economic growth. Specifically, for distinct economic regions in China, the growth of short-term healthcare expenditures will lead to a decline in economic growth, but the corresponding results for the long-term impact of healthcare expenditure on economic growth are significantly different, so hypothesis 3 is tenable. To further test hypothesis 2 and to further explore the impact of health expenditure on economic growth, we simulated the role of the physical and human capital accumulation channels in the transmission process.

There are significant differences between physical capital accumulation and human capital accumulation. Physical capital investment has a faster effect, including it among short-term capital returns. However, human capital investment often has an exceedingly long lag before returns are realized. Whether the returns come through improving national health and then improving effective labor time or through accumulating investment in education for the next generation, they cannot be obtained in a short time. This paper investigates the role of physical and human capital accumulation channels in the economic growth effect of healthcare expenditure in China, Inner Mongolia, Jilin, Beijing, and Tianjin; the results are summarized in Table 4.

**Table 4:** The role of physical capital accumulation and human capital channels in the economic growth effect of healthcare expenditure in China's different regions.

**Regions**	**PCA**		**HCA**	
	**Short-term**	**Long-term**	**Short-term**	**Long-term**
CN	−0.0916	0.0376	0.0356	0.2392
NMG	−0.0075	−0.1277	0.0074	0.0195
JL	−0.0093	−0.0135	0.0067	0.0053
SC	−0.0332	−0.0027	0.0077	0.0164
BJ	−0.0411	0.0565	0.0304	0.0138
YJ	−0.0063	0.0707	0.0031	0.0186

*PCA, represents physical capital accumulation expenditure in GDP (%); HCA, represents human capital accumulation expenditure in GDP (%)*.

As shown in Table 4, in the short term, the material accumulation channel will have a significant negative impact on the economic growth effect of healthcare expenditure, which indicates that increasing healthcare expenditures will enhance the local financial burden in the short term, increase residents' living expenses, and thereby affect consumption, which will significantly inhibit short-term economic growth. Although the accumulation of human capital has a positive effect on the economic growth of healthcare expenditure in the short term, the effect is smaller than that from the accumulation of material capital. There are two reasons leading to the phenomenon in which an increase in healthcare expenditure brings about a decline in short-term economic growth. In the long run, human capital accumulation channels have a positive influence on the effect of healthcare expenditure on economic growth, and this effect is higher than its short-term effect across all economic regions of China. The empirical results are consistent with the economic facts: human capital accumulation tends to take effect in the long run. The impact of material capital accumulation on the effect of healthcare expenditure on economic growth is more complex. The accumulation of material capital in the Inner Mongolia, Jilin and Sichuan regions played a negative role in the long term, with effect ranges of −0.1277, −0.0135, and −0.0027, respectively. All of China, Beijing and Tianjin were positively affected by the accumulation channels of material capital, with effect sizes of 0.0376, 0.0565, and 0.0707, respectively. This illustrates that for the material accumulation channel of China's medical expenditures, economically developed areas will benefit more in the long run because the residents in developed areas have more disposable income, and coverage from the medical security system can reduce family healthcare expenditure in the long run, thus effectively protecting families from poverty caused by illness. At the same time, personal savings can increase more quickly in economically developed areas and then promote economic growth through long-term material accumulation channels. In contrast, in economically underdeveloped areas, fiscal tension, insufficient disposable income and increased healthcare expenditures will crowd out residents' consumption and reduce savings, leading to a decline in long-term economic growth. However, for the whole country, the medical security system is undoubtedly necessary. Although the long-term economic growth of underdeveloped areas will be negatively affected by healthcare expenditures and may need certain “subsidies” from developed areas, healthcare expenditures across the country, whether through the accumulation of physical or human capital, will contribute to long-term economic growth. To a certain extent, healthcare expenditures can help to improve people's welfare and economic efficiency in the long run.

### Robustness Analyses

In this section, we utilize two common robustness test methods to verify the above empirical results. First, the sampling interval is replaced by other subintervals to verify whether the main conclusions we obtained are still valid in other time periods and to try to avoid the influence of the sampling interval on the main research conclusions. Second, by changing the lag order in the SV-TVP-FAVAR model, we can measure the reliability of the above empirical research conclusions from the perspective of the model.

#### Alternative Sample Period

In the baseline analyses, to explore the economic growth effect of national medical and health expenditure, we selected a sampling interval from the first quarter of 2006 to the fourth quarter of 2019; however, the sampling range for analyzing the economic growth effect of medical and health spending in other economic regions of China is limited by the availability of data in each economic region. The effect of health expenditure on economic growth mainly occurs through the accumulation of physical and human capital, but the effect of health expenditure on economic growth is inevitably also affected by other economic variables. To address this issue, this paper introduces the idea of factor enhancement, which abstracts major possible economic variables into three unobservable common factors to include as many factors as possible in the systematic analysis framework. However, the problem is that a longer sampling interval implies that more factors that may ultimately affect the effect of health expenditures on economic growth are included, and the sampling intervals selected in different areas are also diverse. Apart from the sampling interval selected in this paper, does the effect of medical and health expenditure on economic growth still support our main conclusions? To verify the robustness of the empirical research conclusions for other sampling intervals, we choose the first quarter of 2017 to the fourth quarter of 2019 as a new adoption interval that is unified in each region. The verification results are shown in Table 5, and they are analogous to the original conclusions. The results consistently support that the increase in medical and health expenditure is not conducive to short-term economic growth but can benefit long-term economic growth. For other subintervals, such as from the first quarter of 2010 to the fourth quarter of 2012 and from the first quarter of 2015 to the fourth quarter of 2017, we also verify our previous conclusions. Due to the limited space, these results are reserved for retrieval.

**Table 5:** Robustness test results: change in the sampling interval.

	**GDP_S**	**GDP_L**		**GDP_S**	**GDP_L**
HCE_CN	−0.387	1.471	HCE_SC	−0.014	−0.007
HCE_NMG	−0.182	0.659	HCE_BJ	−0.039	0.384
HCE_JL	−0.078	−0.052	HCE_TJ	−0.265	0.515

#### Alternative Selection of Variable Lag Order

**Figure 3:**
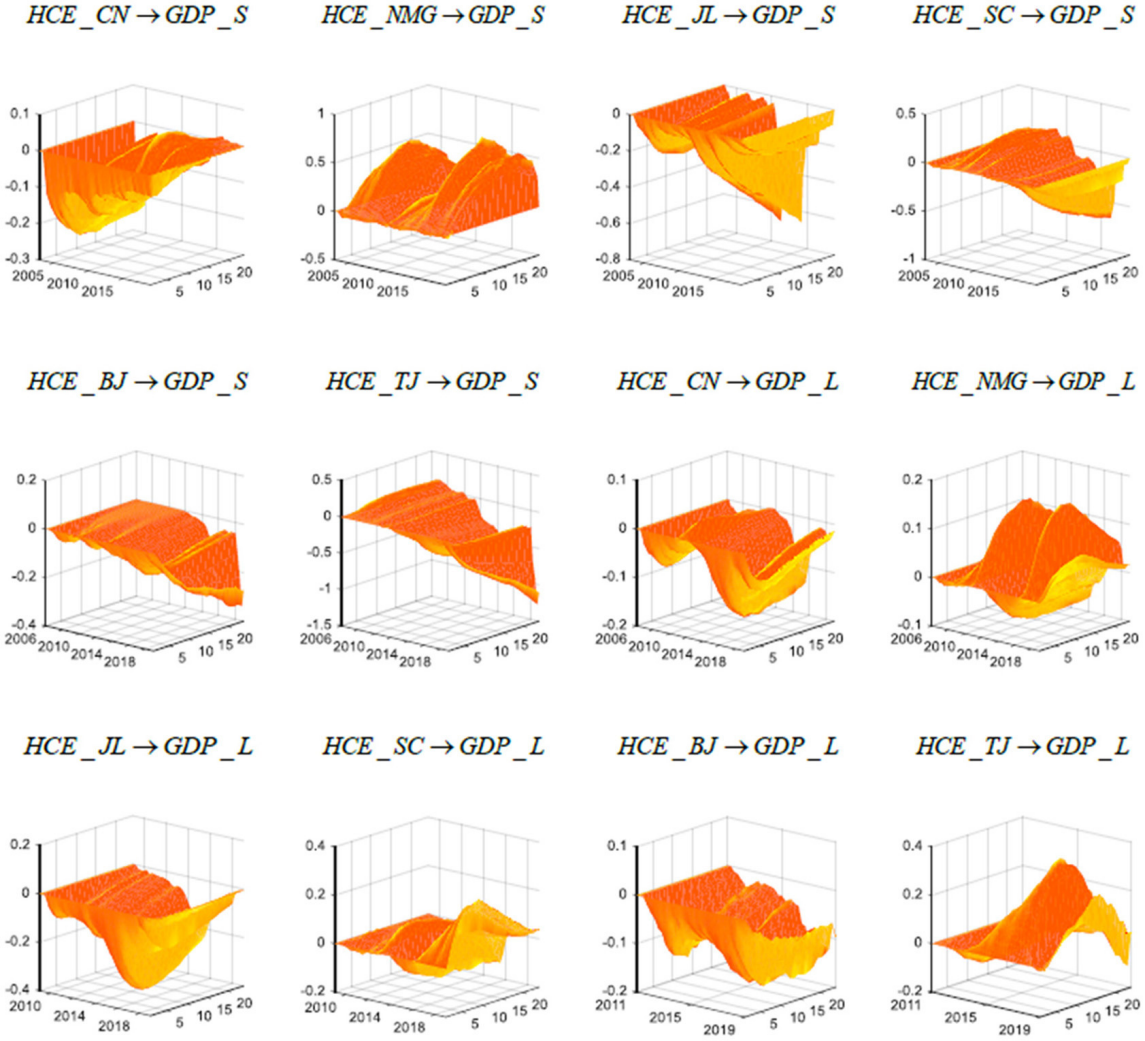
Robustness test results: change in the selection of the variable lag order. The results shown in the figure are for the second-order lag. Due to space limitations, the empirical results of the remaining lagged orders are not listed individually but maintained for reference. Each subfigure with the title of “*X* → *Y*” demonstrates the response of variable Y to an orthogonalized positive shock to variable X. In other words, X is an impulse variable, and Y is a response variable. One period in the figure denotes one season.

In the baseline model, the first-order lag is adopted according to the AIC; thus, in this section, we change the lag order in the model. With the other conditions remaining unaltered, we change the lag order of the model and then use the second-order lag model and the third-order lag model to analyze the effect of medical and health expenditure on economic growth. The results are shown in Figure 3. It can be seen that altering the lag order of the model fails to change the previous empirical results, proving that the conclusions of this paper are robust.

#### Alternative Number of Common Factors

This paper conducts an empirical analysis under a framework that contains many variables related to the effect of China's healthcare expenditure on economic growth. In the factor extraction section, we extracted three common factors. To further verify the robustness of the model test results, we attempt to conduct four and five common factor extractions, and the results show that changing the number of common factor extractions does not change the core conclusion of this article. Due to space limitations, the specific empirical results are not listed but have been maintained for reference.

## Conclusions and Policy Implications

The coverage of the medical security system has a vital impact on people's economic ability to support their lifestyle, and medical and health expenditures also significantly affect the speed of economic growth. However, few scholars have deeply investigated the impact of medical and health expenditures on economic growth in distinct regions of the same country or explored the mechanism behind these effects. By constructing a systematic research framework, this paper analyzes the mechanism through which medical and health expenditures influence economic growth. It offers a comprehensive consideration of potential influencing factors and selects time series data of China and its different economic regions (including Inner Mongolia, Jilin, Sichuan, Beijing, and Tianjin) from the first quarter of 2006 to the fourth quarter of 2019 to empirically test the effect of medical and health expenditure on economic growth and its internal mechanism by using a time-varying parameter model, which can capture slight dynamic changes in the economic system.

The results of the empirical research presented in this paper demonstrate that in China as a whole, increasing medical and health expenditure will produce a negative impact on economic growth in the short term, but in the long term, it will stimulate economic growth. Due to China's vast territory, the level of healthcare and economic development vary greatly for different regions, which will undoubtedly affect how medical and health expenditures influence growth in diverse economic regions within China. When our research focuses on China, we find that there are obvious differences in the effects of medical and health spending on economic growth in different regions. The increase in medical and health expenditures suppresses economic growth in Inner Mongolia and Jilin over the sampling range. It also played a negative role in the economic growth of Sichuan most of the time, though its role has become positive in recent years. Medical and health expenditures play a driving role in the economic growth of Beijing, Tianjin, and other regions, which illustrates that these are a relatively heavy burden on the local finances of subdeveloped areas in China. Due to the fragile economic environment, inadequate infrastructure and other reasons, the benefits brought by the improved medical security system are insufficient to alleviate the pressure on residents and local governments caused by increased expenditures, thereby causing the regional economic growth rate to slow down in the short and long term. For the developed areas in China, due to the high level of infrastructure construction and the high disposable income of residents, medical and health expenditures can provide more convenient health protection for residents. Through the substitution effect, residents can enhance their leisure time for health management and engage in education expenditures to promote investment and consumption, which in turn will benefit the local economy in the long run.

The empirical analysis of the effect of medical and health economic growth also reveals that the long- and short-term effects of medical and health expenditures on economic growth are achieved through the accumulation of physical and human capital. The direction of the effect of the accumulation of physical capital on the growth of medical and health economies is uncertain, but the accumulation of human capital tends to play a positive role in the short and long term. Through the in-depth analysis, it can be seen that an increase in medical and health expenditure in underdeveloped areas (Inner Mongolia, Jilin) in China plays a negative role in short-term economic growth through the channel of material accumulation, while in developed regions (Tianjin, Beijing), that same channel plays the opposite role. The effect of the transmission channel is consistent with the above empirical results in that the economic growth of underdeveloped areas is restrained by an increase in medical expenditure, and the economic growth of developed areas is restrained in the short term but significantly increased in the long term.

Not only China but the whole world is facing the need to balance between “protecting people's livelihood” and “promoting growth,” especially when COVID-19 is spreading globally. Finding a balance between the two with limited resources is a momentous task. The research conclusion of this paper further verifies that the medical security system is a necessity, and its construction as well as its coverage is an important path to ensure people's livelihood and while simultaneously promoting growth. The research illustrates that a sound medical and health security system must be established, producing a positive impact on economic growth in the long run and effectively promoting economic growth through long-term material capital accumulation and human capital accumulation. Substantial economic growth can increase government revenue and provide strong support for the enrichment of livelihood projects. However, there is a major imbalance in China's internal regional development. Therefore, the construction of China's multilevel medical security system cannot be deferred, and certain policy preferences and supporting expenditures are needed for economically underdeveloped areas. The research conclusion of this paper is that a medical security system is irreplaceable for people's livelihood in China, as “ensuring the people's livelihood” is a vital way to promote long-term economic growth; the results affirm the need for the government to spare no effort to treat COVID-19 during the epidemic, motivated by the desire to restore order to the economy as soon as possible, and this need is of great significance to the establishment of a long-term sustainable medical security system in China.

## Data Availability Statement

Publicly available datasets were analyzed in this study. This data can be found at: raw data were generated by the Wind Database. Derived data supporting this study's findings are available from the corresponding author (SW) upon request, without undue reservation.

## Author Contributions

SW: conceptualization, methodology, software, formal analysis, data curation, writing—original draft preparation, writing—review, and editing. BZ, SW, and ZQ: validation. BZ: investigation, resources, supervision, project administration, and funding acquisition. ZQ: visualization. All authors contributed to the article and approved the submitted version.

## Conflict of Interest

The authors declare that the research was conducted in the absence of any commercial or financial relationships that could be construed as a potential conflict of interest.

## Publisher's Note

All claims expressed in this article are solely those of the authors and do not necessarily represent those of their affiliated organizations, or those of the publisher, the editors and the reviewers. Any product that may be evaluated in this article, or claim that may be made by its manufacturer, is not guaranteed or endorsed by the publisher.

## Footnotes

^1^This article also tried to extract other numbers of common factors, but these did not have a significant impact on the empirical results. The results are available on request.

## References

[1] Swift R. The relationship between health and GDP in OECD countries in the very long run. Health Econ. (2011) 20:306–22. 10.1002/hec.1590 20217835

[2] Ye L Zhang X. Nonlinear granger causality between healthcare expenditure and economic growth in the OECD and major developing countries. Int J Environ Res Public Health. (2018) 15:1953. 10.3390/ijerph15091953 30205463 PMC6163394

[3] Baltagi BH Moscone F. Healthcare expenditure and income in the OECD reconsidered: evidence from panel data. Econ Model. (2010) 27:804–11. 10.1016/j.econmod.2009.12.001

[4] Bedir S. Healthcare expenditure and economic growth in developing countries. Adv Econ Bus. (2016) 4:76–86. 10.13189/aeb.2016.040202

[5] Hensher M Tisdell J Canny B Zimitat C. Healthcare and the future of economic growth: exploring alternative perspectives. Health Econ Policy Law. (2020) 15:419–39. 10.1017/s1744133119000276 31685052

[6] Rodríguez AF Valdés MN. Healthcare expenditures and GDP in Latin American and OECD countries: a comparison using a panel cointegration approach. Int J Health Econ Manag. (2019) 19:115–53. 10.1007/s10754-018-9250-3 30267372

[7] Zhu ML Hu Q. The health expenditure and economic growth—an empirical analysis based on regulating effect of medical insurance system. Shanghai J Econ. (2020) 5:P81–95. 10.19626/j.cnki.cn31-1163/f.2020.05.006

[8] An SW Xiong XR. Can regional growth relay drive China's stable economic growth? —based on the analysis of panel data of 285 prefecture-level cities and above. China Soft Sci. (2020) 2:82–93. 10.3969/j.issn.1002-9753.2020.02.008

[9] He FM Chang T Dou ZJ Li F Chang KC. Non-linear impact of china's economic growth on the health of residents-an empirical study based on TVP-FAVAR model. Front Public Health. (2019) 7:380. 10.3389/fpubh.2019.00380 31921745 PMC6933769

[10] Lin W Liu GG Chen G. The Urban Resident Basic Medical Insurance: a landmark reform towards universal coverage in China. Health Econ. (2009) 18(Suppl.2):S83–96. 10.1002/hec.1500 19551750

[11] Si W. Public health insurance and the labor market: evidence from China's Urban Resident Basic Medical Insurance. Health Econ. (2020) 30:403–31. 10.1002/hec.4198 33253447

[12] Sun Z Hou Y. Multi-perspective observation and policy response to regional unbalanced development in China. Manag World. (2019) 35:P1–8. 10.19744/j.cnki.11-1235/f.2019.0101

[13] Wei Y Ma L Wang B. Evaluation and measurement of the economic development differences among the eight comprehensive economic zones in China. J Quant Tech Econ. (2020) 37:P89–108. 10.13653/j.cnki.jqte.2020.06.005

[14] Abdullah SM Siddiqua S Huque R. Is healthcare a necessary or luxury product for Asian countries? An answer using panel approach. Health Econ Rev. (2017) 7:4. 10.1186/s13561-017-0144-8 28124312 PMC5267610

[15] Amiri A Ventelou B. Granger causality between total expenditure on health and GDP in OECD: evidence from the Toda–Yamamoto approach. Econ Lett. (2012) 116:541–4. 10.1016/j.econlet.2012.04.040

[16] Wang KM. Healthcare expenditure and economic growth: quantile panel-type analysis. Econ Model. (2011) 28:1536–49. 10.1016/j.econmod.2011.02.008

[17] Gourdel P Ngoc LH Le Van C Mazamba T. Healthcare and economic growth. Ann Econ Stat. (2004) 75:257–72. 10.2307/20079103

[18] Leung MCM Wang Y. Endogenous healthcare, life expectancy and economic growth. Pac Econ Rev. (2010) 15:11–31. 10.1111/j.1468-0106.2009.00486.x

[19] Strittmatter A Sunde U. Health and economic development—evidence from the introduction of public healthcare. J Popul Econ. (2013) 26:1549–84. 10.1007/s00148-012-0450-8

[20] Yang XS Liu HL An R. Health expenditure, human capital and economic growth: based on analysis of simultaneous equations. Chin Health Econ. (2014) 33:P11–2. 10.7664/CHE20140403

[21] Tao CH Wang YX. The impact of government health expenditure structure on economic growth—based on Lasso regression and panel threshold model. Soft Sci. (2018) 32:P34–8. 10.13956/j.ss.1001-8409.2018.11.08

[22] Li G Liu G Ma S. Resident basic medical insurance and household current consumption. Econ Res J. (2010) A1:P30–8. Available online at: https://kns.cnki.net/kcms/detail/detail.aspx?dbcode=CJFD&dbname=CJFD2010&filename=JJYJ2010S1005&v=O6IUlfr0xi3OP7TIU9JyItLnk6TCl6V1r%25mmd2FxV2HQmTBVMJZvtne%25mmd2FpktLPoAKXH%25mmd2Bbp

[23] Blomqvist AG Carter RA. Is healthcare really a luxury? J Health Econ. (1997) 16:207–29. 10.1016/s0167-6296(96)00534-6 10169095

[24] Feldstein M. International differences in social security and saving. J Public Econ. (1980) 14:225–44. 10.1016/0047-2727(80)90041-9

[25] Feldstein M. Social security and saving: new time series evidence. Natl Tax J. (1996) 49:151–64. 10.3386/w5054

[26] Zhu C Liu XO Lian DX. The impacts of public health insurance on labor market performance. Econ Theory Bus Manag. (2017) 36:P78–89. 10.3969/j.issn.1000-596X.2017.05.006

[27] Jia JX Guo QW Ning J. Traditional cultural beliefs, social security and economic growth. J World Econ. (2011) 8:P3–18. Available online at: https://kns.cnki.net/kcms/detail/detail.aspx?dbcode=CJFD&dbname=CJFD2011&filename=SJJJ201108003&v=PKVIHlLP1q8v27TmV%25mmd2Bi0c6RVbS1nEO3HgPyT7nqKgvBTc%25mmd2FDyr49z9sSkE141e41W

[28] Chou WL. Explaining China's regional health expenditures using LM-type unit root tests. J Health Econ. (2007) 26:682–98. 10.1016/j.jhealeco.2006.12.002 17222930

[29] Clemente J Marcuello C Montañés A Pueyo F. On the international stability of healthcare expenditure functions: are government and private functions similar? J Health Econ. (2004) 23:589–613. 10.1016/j.jhealeco.2003.08.007 15120472

[30] Esteve V Martínez-Zahonero J. Testing the long-run relationship between health expenditures and GDP in the presence of structural change: the case of Spain. Appl Econ Lett. (2007) 14:271–6. 10.1080/13504850500425196

[31] Lorenzoni L Belloni A Sassi F. Health-care expenditure and health policy in the USA versus other high-spending OECD countries. Lancet. (2014) 384:83–92. 10.1016/s0140-6736(14)60571-7 24993914

[32] Lorenzoni L Millar J Sassi F Sutherland D. Cyclical vs. Structural Effects on Healthcare Expenditure Trends in OECD Countries Working. Papers Paris, France: OECD (2017).

[33] Bernanke BS Boivin J. Monetary policy in a data-rich environment. J Monet Econ. (2003) 50:525–46. 10.1016/S0304-3932(03)00024-2

[34] Sims CA. Macroeconomics and reality. Econometrica. (1980) 48:1–48. 10.2307/1912017

[35] Primiceri GE. Time varying structural vector autoregressions and monetary policy. Rev Econ Stud. (2005) 72:821–52. 10.1111/j.1467-937X.2005.00353.x

[36] Nakajima J Kasuya M Watanabe T. Bayesian analysis of time-varying parameter vector autoregressive model for the Japanese economy and monetary policy. J Jpn Int Econ. (2011) 25:225–45. 10.1016/j.jjie.2011.07.004

